# Growth Stage-dependent Bacterial Communities in Soybean Plant Tissues: *Methylorubrum* Transiently Dominated in the Flowering Stage of the Soybean Shoot

**DOI:** 10.1264/jsme2.ME19067

**Published:** 2019-12-27

**Authors:** Shintaro Hara, Masatoshi Matsuda, Kiwamu Minamisawa

**Affiliations:** 1 Graduate School of Life Sciences, Tohoku University 2–2–1 Katahira, Aoba-ku, Sendai 980–8577 Japan; 2 Genesis Research Institute Inc. 4–1–35 Shinmachi, Noritake, Nishi-ku, Nagoya 451–0051 Japan

**Keywords:** *Methylobacterium*, *Methylorubrum*, soybean, 16S rRNA, plant growth stage

## Abstract

Plant-associated bacteria are critical for plant growth and health. However, the effects of plant growth stages on the bacterial community remain unclear. Analyses of the microbiome associated with field-grown soybean revealed a marked shift in the bacterial community during the growth stages. The relative abundance of *Methylorubrum* in the leaf and stem increased from 0.2% to more than 45%, but decreased to approximately 15%, with a peak at the flowering stage at which nitrogen metabolism changed in the soybean plant. These results suggest the significance of a time-series analysis for understanding the relationship between the microbial community and host plant physiology.

To elucidate the mechanisms by which plant-associated microbiomes influence plant growth, the uptake of nutrition, and plant health, reductionistic and repeatable approaches have been developed and include (i) a synthetic community to plant microbial communities in gnotobiotic systems using *Arabidopsis* plants (4, 42, 43), and (ii) genome comparisons of vast numbers of culturable microbes (15, 27). However, microbiome research on field-grown plants is also essential for sustainable agriculture (40). These field studies often dealt with snapshots of microbial communities in terms of the plant growth stage because they focused on environmental factors such as fertilizer levels, crop variety/genotype, and location (1, 5, 11, 22, 23). The physiology and morphology of field-grown plants are reported to markedly change during plant growth stages from seedlings to mature plants for harvesting (6, 46, 47).

A growth stage-dependent community shift in the rhizosphere was recently reported (37, 45); however, the relationships between plant growth and bacterial communities in shoot microbiomes have not yet been elucidated in detail. In the present study, we aimed to clarify whether and the mechanisms by which bacterial communities in soybean plant tissues, including the leaf, stem, root, and pod, change with the growth stages of field-grown soybean plants at frequent intervals.

Seeds of *Glycine max* (cultivar Fukuyutaka) were planted on 23 June 2018 in an experimental field owned by Genesis Research Institute (35.01′16″, 137.07′17″; 15×22 m; Toyota, Aichi, Japan) that had not been used to cultivate any crop in the last 2 years and had cultivated sugarcane (*Saccharum officinarum*) between 2011 and 2015. The field soil was classified as a Gray Lowland (pH [H_2_O], 5.7; total carbon content, 2.2%; NO_3_-N, 4 mg kg^−1^; NH_4_-N, 5 mg kg^−1^; Truog phosphorus content, 38 mg P_2_O_5_ kg^−1^). Before planting, the field was treated with 24 kg N, 200 kg P, and 6.4 kg K as manure and organic materials per hectare. The field was divided into three plots, and each plot was planted with 16 plants (1.3 m between plants, 1.0 m between rows). After every *ca*.10 days (stages S1 to S9), three plants were sampled individually from each plot (Table 1). At 11 and 19 days after sowing (DAS), each plant was divided into the shoot and root because the leaf and stem were too small to divide. At 31, 40, 49, and 61 DAS, plants were divided into the leaf, stem, and root. At 72, 80, and 93 DAS, plants were divided into the leaf, stem, root, and pod. Plant tissues were harvested, washed with tap water, and nodules were removed from root samples by hand picking, weighed, and stored at −20°C until DNA extraction.

Each plant sample was subjected to DNA extraction using a Fast Spin Kit for Soil (MP Biomedicals, Solon, OH, USA) with some modifications (25). Partial 16S rRNA gene sequences were amplified using an Illumina-adapter added primer set specific to the V3–V4 region (341F and 785R, [39]). PCR was performed using the buffer and DNA polymerase system of KOD FX Neo (TOYOBO, Osaka, Japan). The reaction mixture included 12.5 μL PCR buffer, 2 μL template, 0.25 μM of the Illumina-adapter added primer set, and 0.75 μM of each of the two PNAs designed to target host-derived amplicons from chloroplast and mitochondrial 16S rRNA sequences (28). The PCR program was set as follows: 94°C for 2 min, followed by 30 cycles at 98°C for 10 s, 78°C for 10 s, 50°C for 30 s, 68°C for 30 s, and a final extension at 68°C for 5 min. The purification of PCR products and tagging were performed as described previously (19).

Libraries were sequenced on an Illumina Miseq sequencer (300 bp paired-end reads) and demultiplexed. Using QIIME2 pipeline version 2018.11 (10) and dada2 (9), the paired-end fastq files were processed by quality filtering, merging of the paired ends, chimera removal, singleton removal, and construction of a feature table consisting of an amplicon sequence variant (ASV). Reverse reads were truncated to 260 bp using the “–p-trunc-len-r” option implemented in the dada2 plugin due to decreased quality scores of the sequences at the end of the reverse reads. Taxonomy was assigned to ASVs in the feature table with the SILVA database (release 132, [32]) using the Qiime2 feature-classifier plug-in (7), and ASVs classified as chloroplasts or mitochondria were removed. Furthermore, ASVs assigned to the genera *Ishikawaella* and *Rosenbergiella*, which are symbionts in the insect gut and floral nectar (18, 21), were removed from the feature table of the leaf, stem, and pod because they were assumed to cause noise in the analysis of soybean-associated bacteria (Fig. S1). ASVs assigned to the genus *Bradyrhizobium*, a type of rhizobia of soybean, were removed from that of the root (Fig. S2). After quality filtering and feature table construction, 1,409,100 sequences remained present within 2,142–40,624 sequences per sample. The sequence reads of each sample were rarefied to 2,000 reads per sample, and percent relative abundance, alpha diversity, and beta diversity were calculated using the QIIME2 pipeline. All raw sequence data related to the present study are available in the DDBJ Sequence Read Archive (DRA008314).

Table 1 shows the soybean growth stage (14), fresh weight of soybean tissues, and relative growth rate (RGR, [20]), which were calculated from fresh weights (Detailed data in Table S1). RGR values were higher in the vegetative growth stage (S1–S3) than in the seed development stage (S7–S9). The turning point of RGR changes was likely to occur at stage S5, corresponding to the center of the flowering stage.

In leaf and stem tissues, the alpha diversity (Shannon’s diversity index) of the bacterial community was higher at the start and end of sampling stages, and lower at the flowering stage (S4–S6, Fig. 1). In other words, alpha diversity in the leaf and stem decreased during the vegetative growth stage (S1–S3) and increased during the reproductive stage (S7–S9) according to the transition of RGR (Table 1). The alpha diversity of pod tissue showed a similar curve to those of leaf and stem tissues during the seed development stage (S7–S9). On the other hand, the alpha diversity of the root was constantly high throughout all growth stages.

Beta diversity between samples, obtained by calculating the weighted UniFrac distance matrix, is shown with a principal coordinate analysis (PCoA, Fig. S3). The plot of leaf samples showed a linear reciprocating pattern with an inflection at the flowering stage (S4–S6, Fig. S3D). Although the stem sample was plotted on a reciprocating pattern (Fig. S2E), similar to that of the leaf until the flowing stage, its subsequent trajectory appeared to differ from that of leaf tissues. However, no significant differences were observed in pairwise PERMANOVA comparisons between leaf and stem tissues (Fig. S3G). The pattern of the pod was similar to that of the stem (Fig. S3F), whereas the trajectory in root tissues markedly differed from that in other tissues (Fig. S3C).

The relative abundance of *Methylobacterium* markedly changed in shoot tissues, including the leaf and stem, which increased from 2% to more than 45%, and decreased to approximately 30%, with maximum peaks at stage S5 in the leaf and stage S3 in the stem (Fig. 2A, B, and S4). These patterns were consistent with alpha (Fig. 1) and beta diversities (Fig. S3).

*Methylobacterium*, a core microbe in plants, utilizes the C1 compound released from plant tissues and is well-adapted to survive in the phyllosphere (16, 38, 48). Members of *Methylobacterium* were recently re-classified into *Methylobacterium* and *Methylorubrum* based on genomic and phenotypic data (17). Although some members of *Methylorubrum* have been reported as *Methylobacterium* isolated from plants (2, 31, 41), the distribution of *Methylorubrum* remains unclear.

ASVs assigned to the genus *Methylobacterium* were extracted from the feature table. The phylogenetic tree based on 16S rRNA genes was constructed with MEGA version 7 (26); each Methylobacterial ASV was then classified under the criterion of Green and Ardley (17) (Fig. S5). Twenty-two ASVs were classified into *Methylobacterium* clade A (14 ASVs), *Methylobacterium* clade C (6 ASVs), and *Methylorubrum* (2 ASVs). As shown in Fig. 2E, F, and G, ASV0001 (*Methylorubrum*) accounted for the majority, particularly at the flowering stage (S4–S6) in the leaf and stem. Thus, *Methylorubrum* was the dominant bacterium in the soybean shoot, showing a transient time course. In soybean shoots at the early (S1 and S2) and late (S8 and S9) stages, *Gammaproteobacteria* and *Actinobacteria* were abundant instead of *Alphaproteobacteria*, including *Methylobacterium* (Fig. 2A and B), suggesting a negative correlation in their abundance. In the ASV level analysis, only ASVs classified as *Sphingomonas* showed relatively high abundance during the flowering stage (Fig. 2I and S6), even though ASV0001 (*Methylorubrum*) showed high abundance during the flowering stage (S4–S6) in a shoot (Fig. 2I). The genera *Methylobacterium* and *Sphingomonas* are plant-associated bacteria (8, 30), including the soybean leaf (12, 24); however, these findings are just snapshots of the bacterial community. Our time-course experiment suggested the existence of unknown cooperative mechanisms between *Methylobacterium* and *Sphingomonas* in soybean shoot environments.

In the present study, we observed a dynamic bacterial community shift, a particularly transient abundance of *Methylorubrum* in the shoot at the onset of the soybean flowering stage. Since the nitrogen-fixing activity and concentration of nitrogen compounds in xylem sap differ before and after the flowering stage (34, 36, 44), these factors may control the bacterial community shift. Furthermore, heavy nitrogen fertilization reduced the relative abundance of *Methylorubrum* (previous *Methylobacterium extoquance*) and *Methylobacterium* in the soybean shoot at the pod-maturing stage (22, 23). Accordingly, the importance of nitrogen conditions for the bacterial community is suggested. In terms of carbon utilization, the spectra of methylamine and betaine utilization differ in *Methylorubrum* and *Methylobacterium* clade A; all type strains in *Methylorubrum* have the ability to catabolize methylamine, while most strains in *Methylobacterium* clade A do not (17, 29). Similar findings were suggested for betaine (17). Betaine is synthesized in plants and microbes under stress conditions (3). Thus, the compositions of *Methylorubrum* and *Methylobacterium* may provide an important insight into plant-microbe interactions. *Methylorubrum* and *Methylobacterium* (previously *Methylobacterium* group I and groups II & III) were adapted to different plant species of soybean and rice, respectively, in the same field site (29).

As shown in oceans and gut microbial research, a time-series analysis is a useful approach for identifying the stability and dynamics of the microbial community (13, 33, 35). The combination of a time-series analysis of the microbial community and host physiology may provide novel insights into the physiological relationships between plants and the microbiome.

## SUPPLEMENTARY MATERIAL



## Figures and Tables

**Fig. 1:**
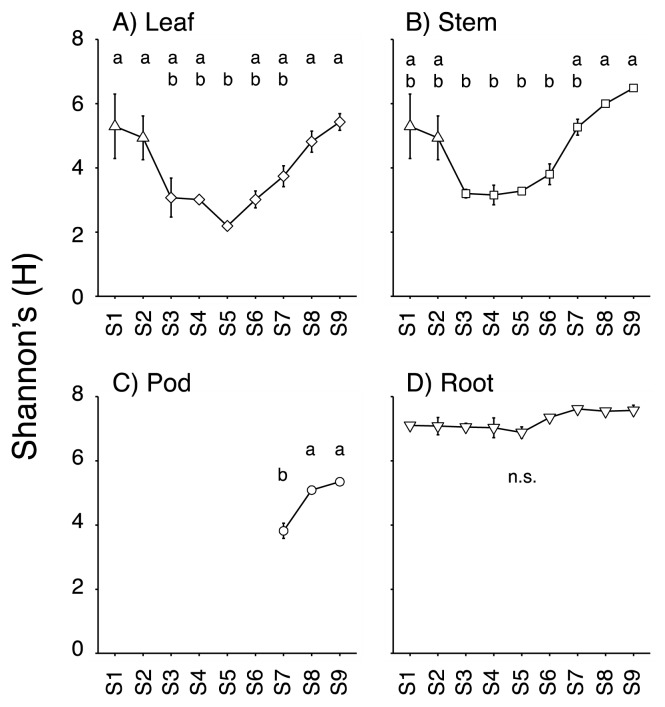
Mean Shannon’s diversity index of leaf (A), stem (B), pod (C), and root (D) microbes at each stage. Error bars represent the standard error from the mean (*n*=3). Different letters indicate a significant difference as assessed by Tukey’s HSD test (*P*<0.05). n.s., not significant.

**Fig. 2:**
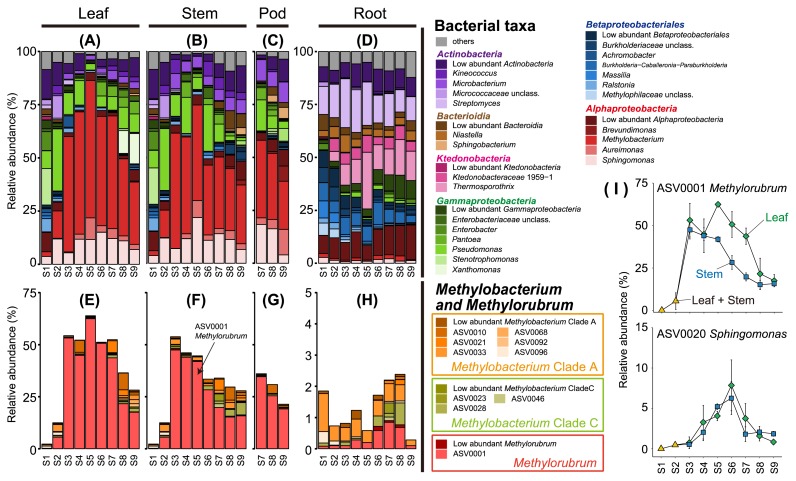
Fluctuations in the bacterial community in soybean tissue depend on the growth stage. All values represent the mean of triplicates. In leaf and stem samples, the same value is shown at stages S1 and S2. (A, B, C, and D) The relative abundance of the genus from all bacterial taxa. Individual values of replicates are shown in Fig. S4. (E, F, G, and H) The relative abundance of the amplicon sequence variance (ASV) assigned to *Methylobacterium* in the Silva database (32). (I) The relative abundance of ASV0001 (*Methylorubrum*) and ASV0020 (*Sphingomonas*) in the leaf and shoot. Error bars represent the standard error.

**Table 1:** Fresh weight and growth rate of soybean tissues with growth stages

Stage	Day after sowing	Growth stage^a^		Fresh weight (g)^b^				Relative growth rate (g g^−1^ d^−1^)^b^^c^		

				Leaf	Stem	Pod	Root			
S1	11	VC	V	2.4 d			1.3	S1–S2	0.11	ab
S2	19	V3		5.7 d			2.7	S2–S3	0.17	a
S3	31	V7		26.8	20.4		8.3	S3–S4	0.17	a
							
S4	40	R1	F	105.2	111.9		24.7	S4–S5	0.13	a
S5	49	R2		278.2	387.8		51.2	S5–S6	0.05	bc
S6	61	R2		518.0	756.0		81.0	S6–S7	0.04	bc
							
S7	72	R3	S	782.3	1223.3	34.0	117.3	S7–S8	0.02	c
S8	80	R4		809.3	1303.3	332.0	176.7	S8–S9	0.02	c
S9	93	R5		826.3	1402.7	898.0	120.0			

a Growth stage of soybean (14); VC, unifoliolate; V3, 3rd trifoliolate; V7, 7th trifoliolate; R1, beginning bloom; R2, Full bloom; R3, beginning pod; R4, full pod; R5, beginning seed; V, F, and S indicate vegetative growth, flowering, and seed development, respectively.

b Mean value of triplicates. The standard error is shown in Table S1.

c Different letters indicate significant differences as assessed by Tukey’s HSD test (*P*<0.05).

d The leaf and stem were treated as one sample at growth stages S1 and S2.
