# Supplementary material for: Growth Stage-dependent Bacterial Communities in Soybean Plant Tissues: Methylorubrum Transiently Dominated in the Flowering Stage of the Soybean Shoot
Source: Microbes Environ. 2019 Dec 27;34(4):446–50. doi: 10.1264/jsme2.ME19067 (PMC6934392; doi:10.1264/jsme2.ME19067)
Supplement: Supplementary file 1 [file 34_446_s1.pdf]

Supplemental material

Growth Stage-dependent Bacterial Communities in Soybean Plant Tissues: *Methylorubrum* Transiently Dominated in the Flowering Stage of the Soybean Shoot

Shintaro Hara[1]*, Masatoshi Matsuda[2], and Kiwamu Minamisawa[1]

[1]*Graduate School of Life Sciences, Tohoku University, 2–2–1 Katahira, Aoba-ku, Sendai 980–8577, Japan*

[2]*Genesis Research Institute Inc., 4-1-35 Shinmachi, Noritake, Nishi-ku, Nagoya 451-0051, Japan*

* Corresponding author. E-mail: shintaro.hara.b4@tohoku.ac.jp, Tel/Fax: +81 022-217-5687

Table S1. Mean fresh weight with error bar and growth rate of soybean tissues during growing stages.

Stage	Day after sowing	Growth stage [a]		Fresh weight (g) [b]					Relative growth rate (g g^{-1} d^{-1}) [bc]		
				Leaf	Stem	Pod	Shoot (Leaf + Stem + Pod)	Root	Shoot (Leaf + Stem + Pod)		
S1	11	VC	V	2.4 ± 0.3 [d]			2.4 ± 0.3	1.3 ± 0.1	S1–S2	0.11 ± 0.03	ab
S2	19	V3		5.7 ± 1.2 [d]			5.7 ± 1.2	2.7 ± 0.5	S2–S3	0.17 ± 0.01	a
S3	31	V7		26.8 ± 8.1	20.4 ± 6.7		47.2 ± 14.9	8.3 ± 1.9	S3–S4	0.17 ± 0.02	a
S4	40	R1	F	105.2 ± 20.5	111.9 ± 27.9		217.1 ± 48.3	24.7 ± 1.6	S4–S5	0.13 ± 0.01	a
S5	49	R2		278.2 ± 32.2	387.8 ± 61.6		666.0 ± 93.8	51.2 ± 3.2	S5–S6	0.05 ± 0.00	bc
S6	61	R2		518.0 ± 60.0	756.0 ± 86.2		1274.0 ± 142.2	81.0 ± 2.6	S6–S7	0.04 ± 0.01	bc
S7	72	R3	S	782.3 ± 52.1	1223.3 ± 87.8	34.0 ± 4.2	2039.7 ± 132.5	117.3 ± 4.7	S7–S8	0.02 ± 0.01	c
S8	80	R4		809.3 ± 26.2	1303.3 ± 80.2	332.0 ± 45.7	2444.7 ± 99.2	176.7 ± 69.7	S8–S9	0.02 ± 0.01	c
S9	93	R5		826.3 ± 48.0	1402.7 ± 53.9	898.0 ± 41.6	3127.0 ± 126.4	120.0 ± 8.1			

[a] Growth stage of soybean (Fehr, 1971); VC, unifoliolate; V3, 3rd trifoliolate; V7, 7th trifoliolate; R1, beginning bloom; R2, Full bloom; R3, beginning pod; R4, full pod; R5, beginning seed; V, F, and S indicate vegetative growth, flowering, and seed development, respectively.

[b] Mean value with standard error (n=3).

[c] Different letters indicate significant differences as assessed by Tukey's HSD test (P <0.05).

[d] Leaf and stem were treated as one tissue at S1 and S2.

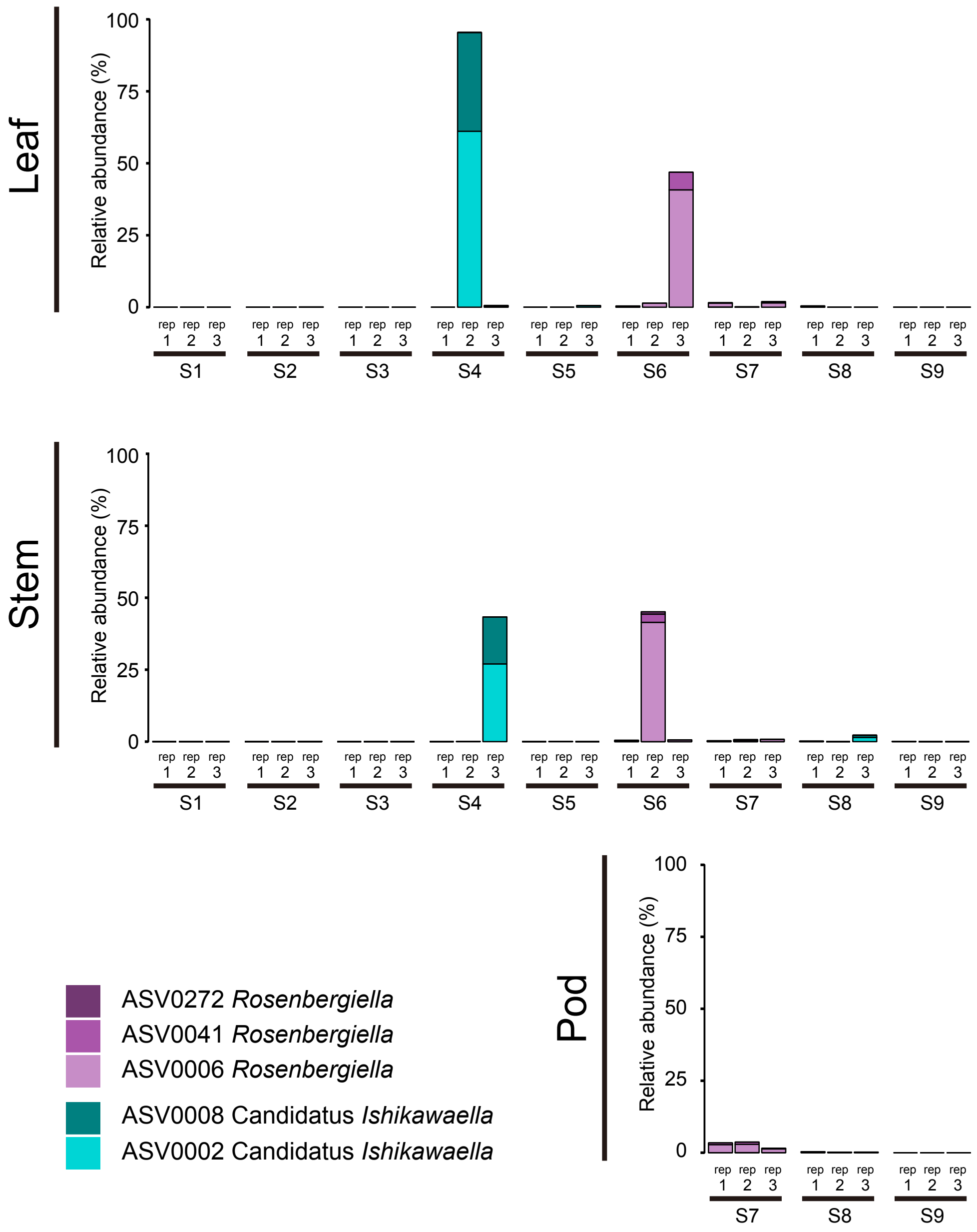

Figure S1. Relative abundance of amplicon sequence variances assigned to *Rosenbergiella* and *Ishikawaella*.

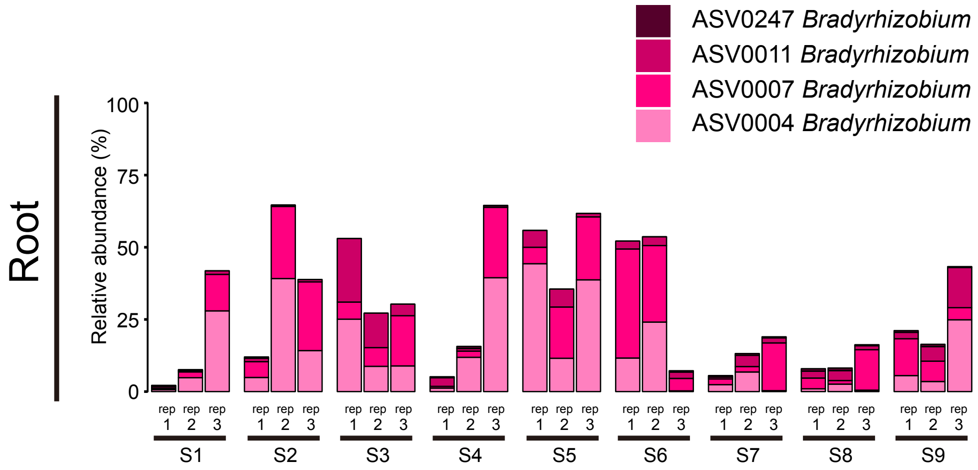

Figure S2. Relative abundance of amplicon sequence variances assigned to *Bradyrhizobium*.

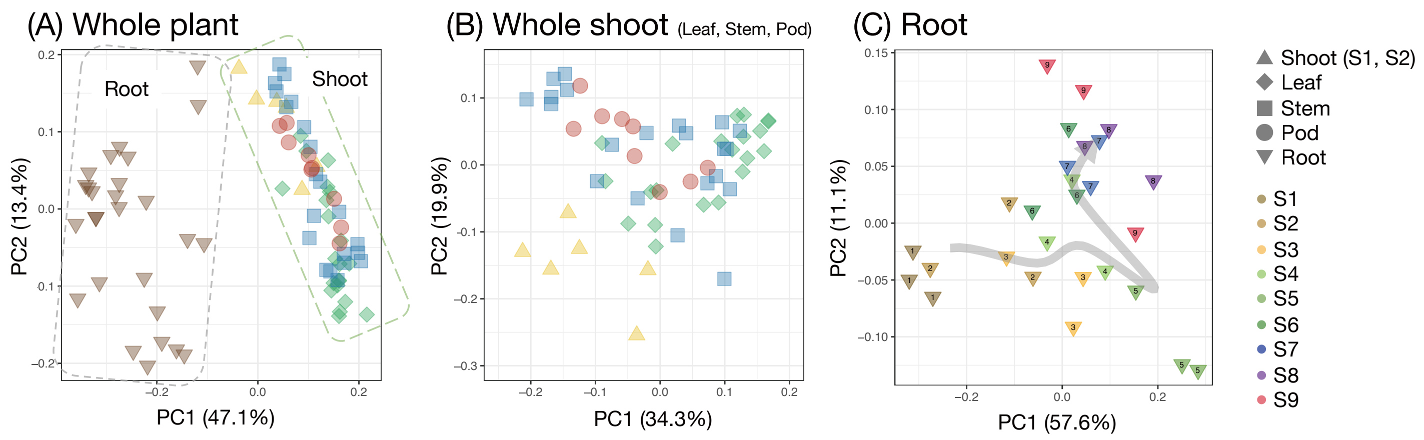

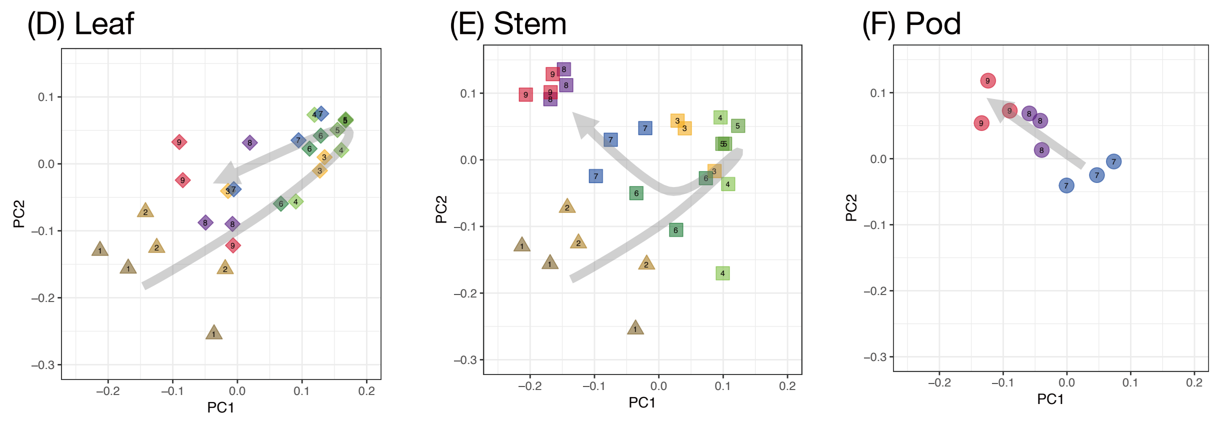

(G) Permanova
(Leaf vs Stem)

	p value
S3	0.620
S4	0.511
S5	0.090
S6	0.104
S7	0.199
S8	0.105
S9	0.084

Figure S3. Principal Coordinate analysis of weighted UniFrac distance matrix showing the transition of the bacterial community in soybean plant depends on the growth stage. (A) All samples were analyzed together, and two clusters representing shoot (leaf, stem, and pod) and root were observed. Shoot (B) and root (C) samples were re-analyzed independently. Samples of leaf (D), stem (E), and pod (F) were collected and shown individually. (G) Permutational multivariate analyses of variance (PERMANOVA) with 999 permutations were performed between leaf and stem samples at each stage.

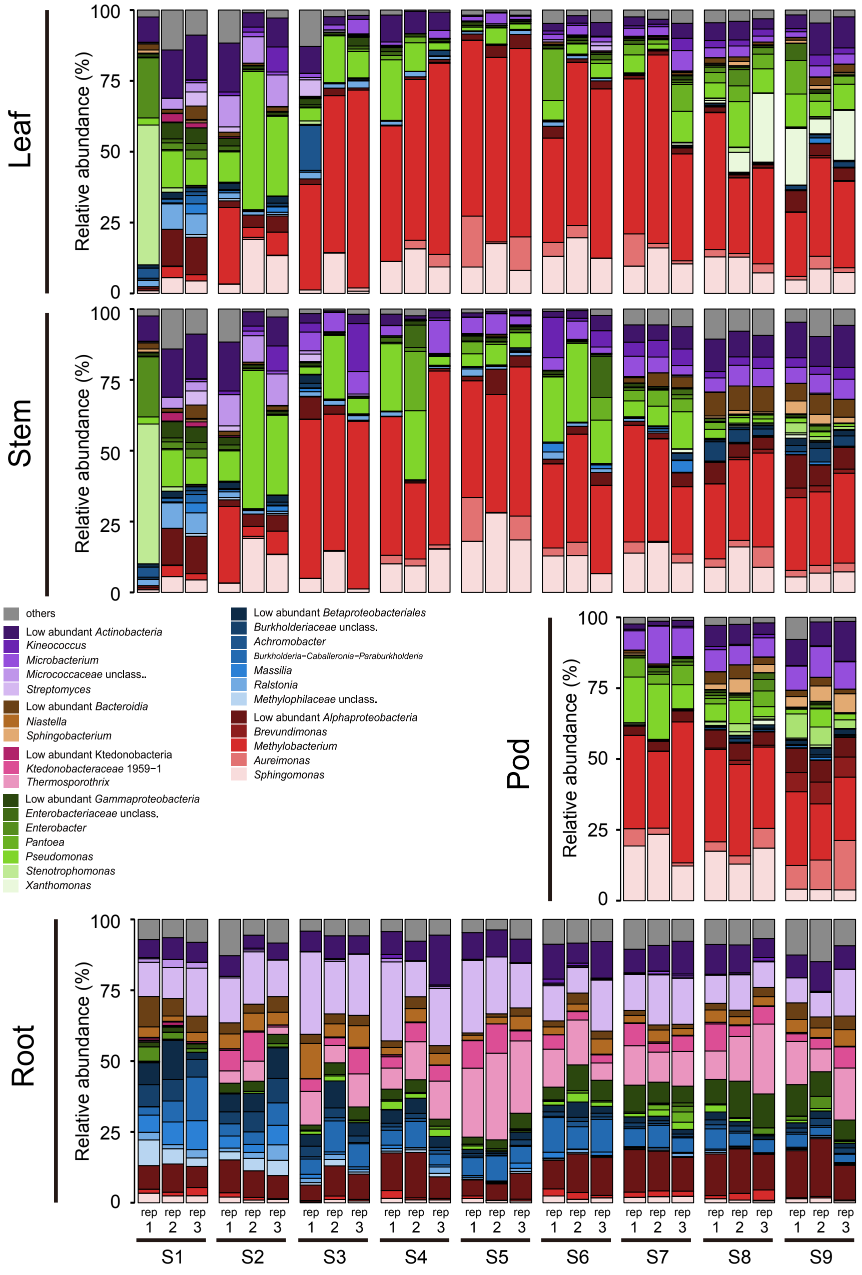

Figure S4. Relative abundance of the genus from all bacterial taxa with individual values of triplicates.

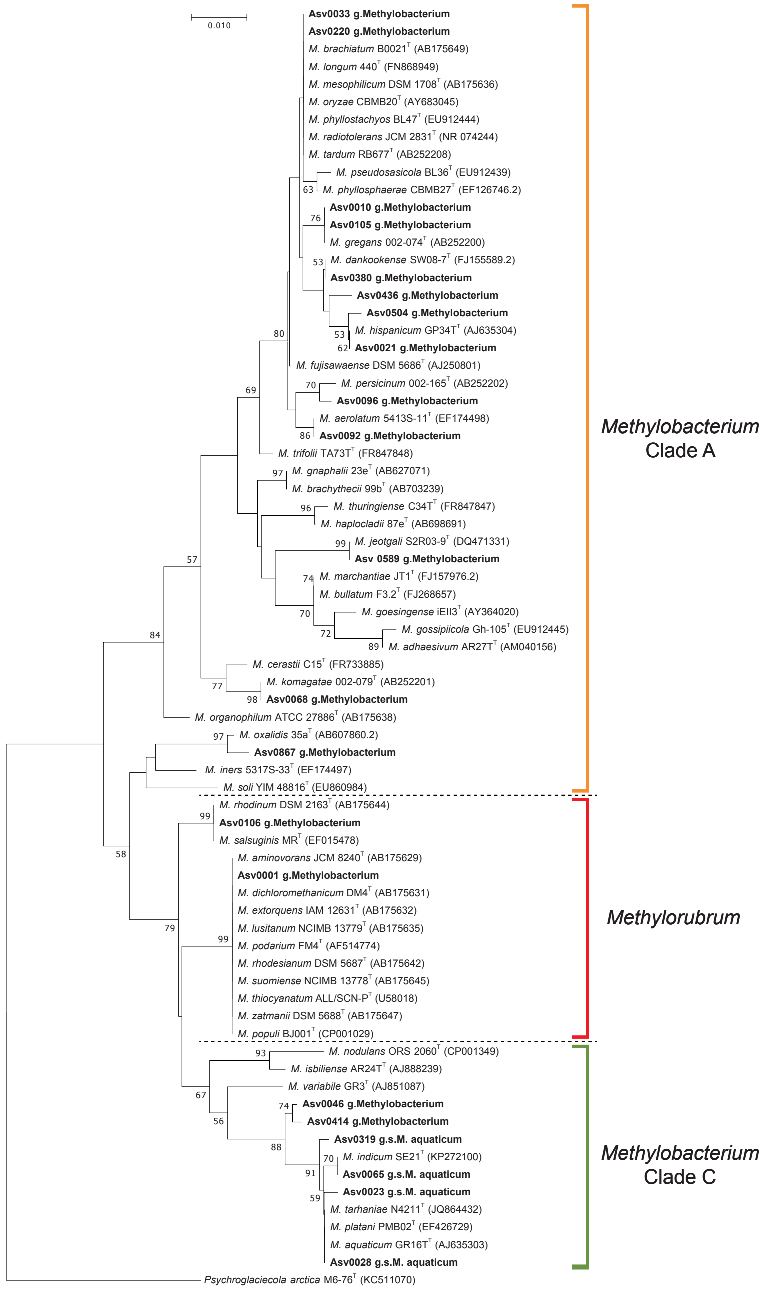

Figure S5. Phylogenetic tree based on 16S rRNA gene sequences showing the taxonomic positions of Methylobacterial amplicon sequence variance (ASVs) constructed by the neighbor-joining method. The ASVs detected in this study are given in bold. Taxa were identified to the lowest possible classification with SILVA database (release 132; Quast et al., 2013) , where g. is genus and s. is species. The type strains of *Methylobacterium* were used as references (Green and Ardley, 2018). Bootstrap values were analyzed with 1000 replicates, and the values more than 50 % are indicated at nodes.

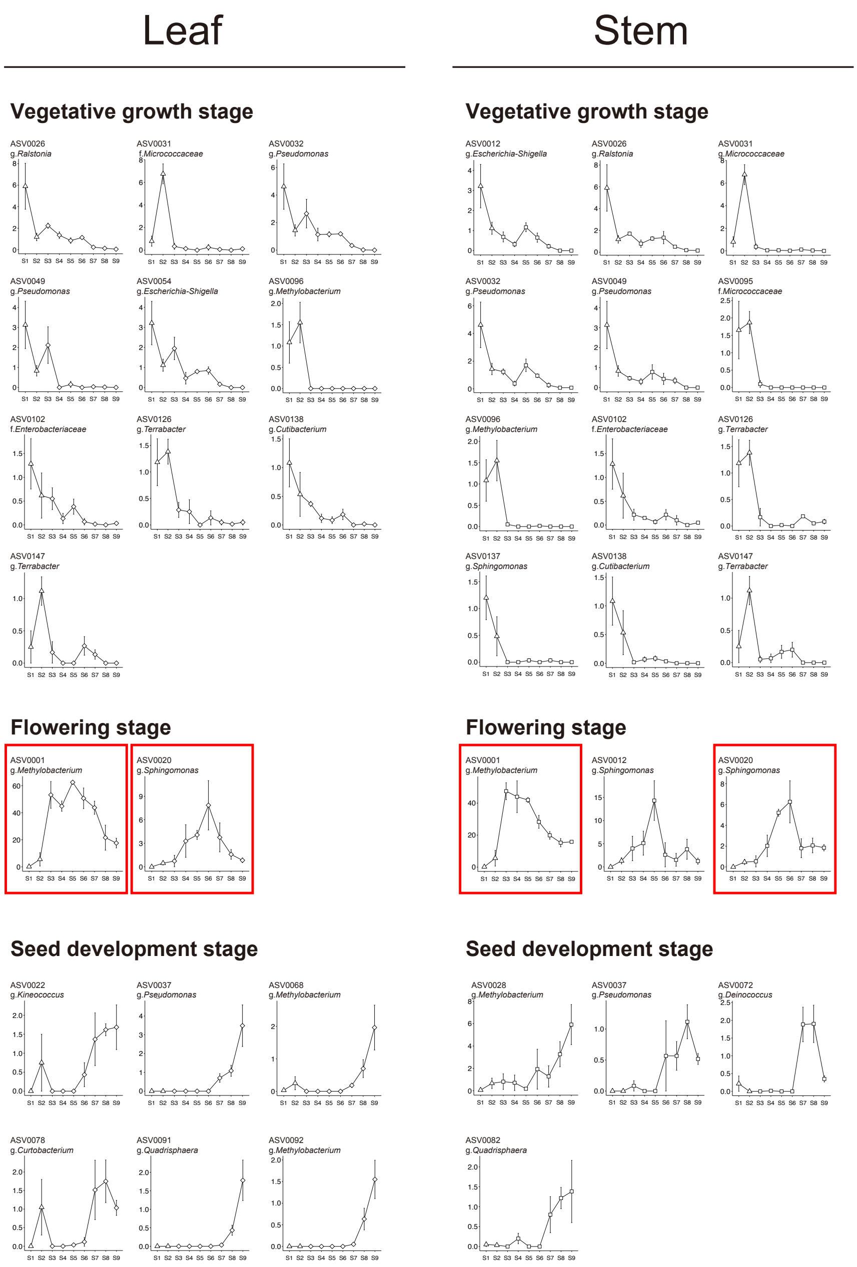

Figure S6. A transient pattern of relative abundance of amplicon sequence variances (ASVs). The ASVs affected by sampling stage (one-way ANOVA, P<0.05) were retrieved and shown with groups having the highest mean abundance in the vegetative growth stage, flowering stage, or seed development stage. The values on X-axis show the sampling stage, and those on Y-axis show relative abundance (%). Error bars represent standard error from the mean (n=3). The red boxes indicate ASVs shown in Fig. 2I.

References

Fehr, W.R. 1971. Stage of Development Descriptions for Soybeans, Glycine Max (L.) Merrill. Crop Sci. 11:929–931.

Green, P.N., and J.K. Ardley. 2018. Review of the genus *Methylobacterium* and closely related organisms: A proposal that some *Methylobacterium* species be reclassified into a new genus, *Methylorubrum* gen. nov. Int. J. Syst. Evol. Microbiol. 68:2727–2748.

Hoffmann, W.A., and H. Poorter. 2002. Avoiding bias in calculations of relative growth rate. Ann. Bot. 90:37–42.

Quast, C., E. Pruesse, P. Yilmaz, J. Gerken, T. Schweer, P. Yarza, J. Peplies, and F.O. Glöckner. 2013. The SILVA ribosomal RNA gene database project: Improved data processing and web-based tools. Nucleic Acids Res. 41:590–596.
